# Animal-based remedies as complementary medicines in Santa Cruz do Capibaribe, Brazil

**DOI:** 10.1186/1472-6882-8-44

**Published:** 2008-07-22

**Authors:** Rômulo RN Alves, Helenice N Lima, Marília C Tavares, Wedson MS Souto, Raynner RD Barboza, Alexandre Vasconcellos

**Affiliations:** 1 Departamento de Biologia, Universidade Estadual da Paraíba, Avenida das Baraúnas, Campina Grande, Paraíba 58109-753, Brazil; 2 Programa de Pós-Graduação em Desenvolvimento e Meio Ambiente (PRODEMA), Universidade Estadual da Paraíba, Avenida das Baraúnas, Campina Grande, Paraíba 58109-753, Brazil; 3 Mestrado em Ciência e Tecnologia Ambiental, Universidade Estadual da Paraíba, Avenida das Baraúnas, Campina Grande, Paraíba 58109-753, Brazil; 4 Departamento de Botânica, Ecologia e Zoologia, Universidade Federal do Rio Grande do Norte, Natal, RN 59072-900, Brazil

## Abstract

**Background:**

The use of animal products in healing is an ancient and widespread cross-cultural practice. In northeastern Brazil, especially in the semi-arid region, animals and plants are widely used in traditional medicine and play significant roles in healing practices. Zootherapies form an integral part of these cultures, and information about animals is passed from generation to generation through oral folklore. Nevertheless, studies on medicinal animals are still scarce in northeastern Brazil, especially when compared to those focusing on medicinal plants. This paper examines the use and commercialization of animals for medicinal purposes in Brazil's semi-arid *caatinga *region.

**Methods:**

Data was obtained through field surveys conducted in the public markets in the city of Santa Cruz do Capibaribe, Pernambuco State, Brazil. We interviewed 16 merchants (9 men and 7 women) who provided information regarding folk remedies based on animal products.

**Results:**

A total of 37 animal species (29 families), distributed among 7 taxonomic categories were found to be used to treat 51 different ailments. The most frequently cited treatments focused on the respiratory system, and were mainly related to problems with asthma. Zootherapeutic products are prescribed as single drugs or are mixed with other ingredients. Mixtures may include several to many more valuable medicinal animals added to other larger doses of more common medicinal animals and plants. The uses of certain medicinal animals are associated with popular local beliefs known as 'simpatias'. We identified 2 medicinal species (*Struthio camelus *and *Nasutitermes macrocephalus*) not previously documented for Brazil. The use of animals as remedies in the area surveyed is associated with socio economic and cultural factors. Some of the medicinal animal species encountered in this study are included in lists of endangered species.

**Conclusion:**

Our results demonstrate that a large variety of animals are used in traditional medicinal practices in Brazil's semi-arid northeastern region. In addition to the need for pharmacological investigations in order to confirm the efficiency of these folk medicines, the present study emphasizes the importance of establishing conservation priorities and sustainable production of the various medicinal animals used. The local fauna, folk culture, and monetary value of these activities are key factors influencing the use and commercialization of animal species for therapeutic purposes.

## Background

Plants and animals have been used as medicinal sources since ancient times [1-4], and even today animal and plant-based pharmacopeias continue to play an essential role in world health care [5]. The use of biological resources for various therapies has been documented in many different parts of the world – but largely in remote regions, where traditional medicines provide a *de facto *alternative to "modern" health care systems [6-12]. Recent studies, however, have highlighted the relevant role also played by traditional medicine in urban areas [13-19] where health care needs are generally met by mainstream services such as hospitals and allopathic pharmacies [16].

Although plants and plant-derived materials make up the majority of ingredients used in most traditional medical systems, whole animals, animal parts, and animal-derived products (e.g., urine, fat, etc.) also constitute important elements of the folk pharmacopoeia throughout the world. Indeed, zootherapy (the use of animal products in healing) is an ancient and widespread practice across most cultures [3,20]. Traditional medicine still makes use of animals and products derived from animal organs [1], and examples of the use of animal-derived remedies can currently be found in many urban, semi-urban, and more remote localities in all parts of the world [9,10,21-26].

Biological remedies are openly commercialized in essentially all of the towns and cities in Brazil, principally in public markets. It is common to find specific places in these markets where plants and animals are sold for medicinal purposes – locations that serve to unite, maintain, and diffuse empirical knowledge from different regions and of different origins [11,13,16,18,19,22,24,25,27-30]. The on-going search for natural products, as part of a collective social strategy, emphasizes the importance of these traditional centers. However, despite their cultural relevance, few ethnobotanical and ethno-zoological studies have focused on herbal vendors in public and/or open markets [9,31]. Almeida and Albuquerque [22] have pointed out that the information obtained in these markets concerning the exotic and the native flora and fauna may aid in formulating conservation strategies for commercialized natural resources.

In northeastern Brazil, especially in the semi-arid region, animals and plants are widely used in traditional medicine and play a significant role in healing practices there [9,10,16,24]. Zootherapies form an integral part of the local culture, and information about animals and their uses are passed from generation to generation through oral folk lore. Studies on medicinal animals, however, are still scarce when compared to those focusing on medicinal plants. The present work therefore sought to contribute to our knowledge of the medicinal animals used by the inhabitants of the *caatinga *region in northeastern Brazil. The *caatinga *is a highly threatened biome covering a vast area of Brazil, and is the source of many little-known natural resources [32,33]. About 15% of the Brazilian population (more than 25 million people [34] lives in the *caatinga *region and the rural populations there are characterized by extreme poverty [35]. Because of the adverse environmental condition in the region the local populations have developed unique social-environmental structures as well as strong relationships with the natural resources available in the region, including those used for medicinal purposes.

The use of medicinal plants and animals in the semi-arid region has a strong relation with socioeconomic factors, as a large part of the human populations living there do not have access to adequate health services and use phytotherapeutic and zootherapeutic products as easily accessible and low-cost alternatives to medicines sold in commercial pharmacies [11,36]. The gathering and selling of these products is also compatible with popular traditions, and can even generate some income.

In this context, the present study surveyed the medicinal animals sold in public markets in Santa Cruz do Capibaribe, Pernambuco State, in the semi-arid region of northeastern Brazil. The study primarily focused on field surveys to address the following questions: which animal species are used for medicinal purposes? Which animal body parts are used to prepare these remedies? What are the illnesses treated by these remedies? By highlighting the role played by animal-based remedies in Brazil's semi-arid northeastern region we hope to increase awareness about zootherapeutic practices and contribute to the conservation of both cultural and biological diversity.

## Methods

### Study site

**Figure 1:**
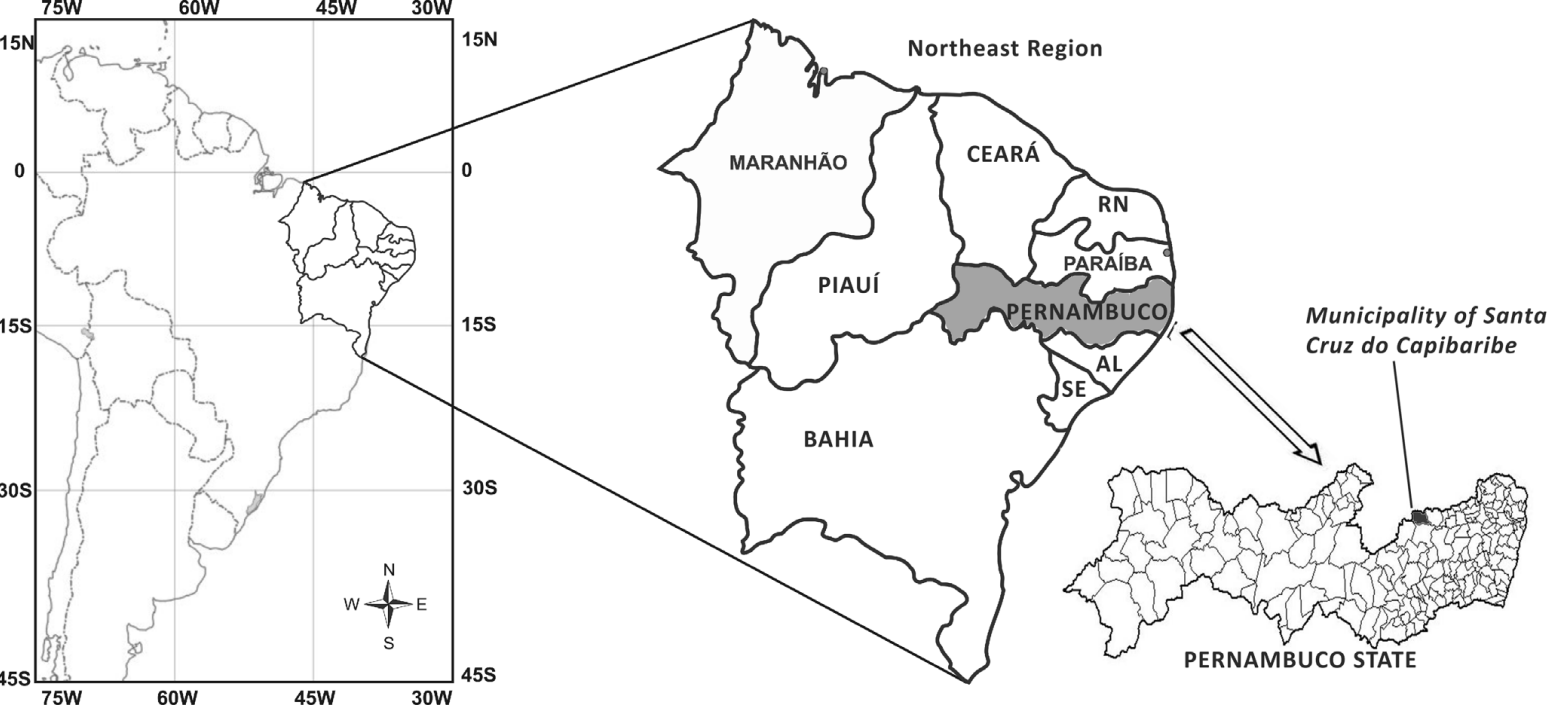
Map of study area, Municipality of Santa Cruz do Capibaribe, Brazil.

The municipal district of Santa Cruz do Capibaribe (07° 57' 27" S × 36° 12' 17" W) is located in Pernambuco State, Brazil, in the microregion of Alto do Capibaribe (Figure 1). The municipality has an area of about 336 km^2 ^and is located 194 km from the state capital of Recife. According to the Brazilian Institute of Geography and Statistics (IBGE), the population there was 59,048 inhabitants in 2000, with 57, 226 living in the urban zone, and 1,822 in rural areas. Recent data indicates that the population has increased to about 73,700 inhabitants [37], however, and according to the IBGE this city grew more than any other in the state during the last ten years. The principal economic activities in the municipality are manufacturing and commerce, with a major potential for growth in garment production. The health services in the municipality include two hospitals and 32 Community Health Agents. According to the Brazilian Ministry of Mines and Energy (MME) the regional vegetation is basically composed of hyperxerophilous *caatinga*, with some remnant patches of deciduous forest. The climate there is tropical semi-arid with summer rains [38]. The city is located in the Capibaribe River basin (the most important in Pernambuco State).

### Procedures

Fieldwork was carried out during the period from May 2007 to October 2007, when public markets were visited in Santa Cruz do Capibaribe. We interviewed 16 merchants (9 men and 7 women) about the use and commercialization of medicinal animals (essentially all of the merchants dealing with animals for medicinal purposes in the city). The sampling method was non-random, and the interviewees were pre-selected [39]. The interviewees ranged in age from 46 to 65 years (average age 56) and all had low degrees of formal education. Essentially all of the interviewees acknowledged earning the Brazilian minimum wage (R$ 380 = USD 211) or less. The collected data through semi-structured interviews was complemented by free-interviews [40], and the informants were requested to furnish the vernacular name, folk use, parts used, and the mode of preparation as well as administration of the remedies made from each type of animal they sold. The ethical approval for the study was obtained from the Ethics committee of Paraiba University State.

Vernacular names of the species cited were carefully noted during the interviews. Zoological material was identified with the aid of specialists through (a) the examination of voucher specimens donated by the interviewees or purchased at the surveyed markets, or (b) photographs of the animal species or their parts taken during interviews. Whenever necessary, procedures (a) and (b) were supplemented by checking the vernacular names provided by the shop owners against scientific names indicated by taxonomists familiar with the study area. Voucher specimens and/or photographs were deposited with the Department of Systematics and Ecology, Universidade Federal da Paraíba.

## Results and Discussion

Animals have been used for medicinal purposes since colonial times in Brazil [11] and they still play a significant role in current folk healing practices. Various publications have shown the importance of zootherapy to traditional communities in various socio-cultural environments in Brazil [11,41-44], and the commercialization of the medicinal animals in various Brazilian cities has been documented by numerous authors [16,18,22,24,45,46].

In Santa Cruz do Capibaribe, as in other cities in Brazil, plants and animals are sold for medicinal purposes in traditional public markets. Table 1 summarizes the names (vernacular and scientific) of medicinal animals, the parts used, ailments treated, and the mode of preparation and/or use. A total of 37 species distributed among 29 families were reported as having medicinal value by the shop owners. The taxonomic group with the largest number of animal species was insects (with 10 species), followed by birds (9), mammals (8), and reptiles (6). Other groups mentioned by the interviewees were fish (2), echinoderms (1), and amphibians (1).

**Table 1:** Animals species used in popular medicine in the municipality of Santa Cruz do Capibaribe, Pernambuco State, Brazil.

**Famíly/species/local name**	**Number of citation**	**Part used and mode of administration**	**Disease (or illness)**
***Insects***			
Apidae			
*Apis mellifera *(Linnaeus, 1758) – Africanised honey bee, "abelha italiana"	1	Honey (3)	Cough, catarrh
*Frieseomelitta varia *(Lepeletier, 1836) – Bee, "Abelha moça branca"	1	Honey (4)	Gonorrhea
*Melipona scutellaris *(Latreille, 1811) – a stingless bee, "uruçú"	3	Honey (3, 4)	Fatigue (3, 4), Cancer (3), Weakness (3), Sexual problems (3), Cough (3)
*Partamona cupira *(Smith) – a stingless Bee, "Abelha cupira"	3	Honey (1, 4), Bee wax (11)	Leucoma, "Slightly clean", Cuts, Wounds, Cough, Catarrh, "toady" (Fungal buccal Infection), Sinusitis, Effusion (11)
*Scaptotrigona *sp. – Bee, "Abelha canudo"	1	Honey (3)	Hernia
*Tetragonisca angustula *Latreille, 1811 – Bee, "Abelha jati"	1	Honey (1)	"Ervilhida" (Leucoma)
*Trigona spinipes *(Fabricius, 1793) – a stingless bee, "arapuá	1	Honey (4)	Fatigue, effusion
Termitidae			
*Nasutitermes macrocephalus *(Silvestri, 1903) – Termite, "Cupim de aroeira"	1	Whole animal (3)	Asthma
Blattidae			
*Periplaneta americana *(Linnaeus, 1758) – American cockroach, "barata"	1	Whole animal (17)	Avoiding pregnancy
Muscidae			
*Musca domestica *(Linnaeus, 1758) – House fly, "Mosca"	1	Whole animal (16)	Hair erysipelas

***Echinoderms***			
Oreasteridae			
*Oreaster reticulatus *(Linnaeus, 1758) – Starfish, "estrela-do-mar"	1	Whole animal (12)	Asthma
*Fish*			
Erythrinidae			
*Hoplias malabaricus *(Bloch, 1794) – Trahira, "traíra	1	Fat (4)	Sore throat
Syngnathidae			
*Hippocampus reidi *(Ginsburg, 1933) – Longsnout seahorse, "cavalo-marinho"	2	Whole animal (12, 19)	Asthma

***Amphibians***			
Bufonidae			
*Rhinella jimi *(Stevaux, 2002)	2	Secretions (14), Fat (4)	Gastritis (14), cancer (4, 14)

***Reptiles***			
Alligatoridae			
*Caiman latirostris *(Daudin, 1801) – Cayman, "jacaré-do-papo-amarelo"	1	Leather (13)	Epilepsy
Chelidae			
*Phrynops geoffroanus *(Schweigger, 1812) – Geoffroy's side-necked turtle, "cágado"	5	Fat (1)	In-grown nail, earache, eczema, articulation problems, wounds
Iguanidae			
*Iguana iguana *(Linnaeus, 1758) – Common Iguana, "Camaleão"	2	Fat (1)	Body aches, wounds
Teiidae			
*Tupinambis merianae *(Duméril & Bibron, 1839) – Teju lizard, "tegu", "tejuaçú"	5	Fat (1, 4)	Wounds (1), sore throat (1,4), earache (1), perforation (1)
Testudinidae			
*Chelonoidis denticulata *(Linnaeus, 1766) – Yellow-footed tortoise, "jabuti"	1	Urine (1), Fat (4)	Ear ache (1), asthma (4), pains (1)
Viperidae			
*Crotalus durissus *Linnaeus, 1758 – South American rattlesnake, "Cascavel"	6	Fat (1, 4)	Rheumatism, swellings, Cancer, Bone aches, Gastritis, eczema, wounds, backache

***Birds***			
Anatidae			
*Anas platyrhynchos *Linnaeus, 1758 – Mallard, "Pata"	1	Eggs (6)	General weaknesses, Sexual weakness, nervous disturbances
Ciconiidae			
*Coragyps atratus *(Bechstein, 1793) – Black vulture, "Urubu"	1	Feather and liver (15)	Alcoholism
Corvidae			
*Cyanocorax cyanopogon *(Wied-Neuwied, 1821) – White-naped Jay, "Pássaro cancão"	1	Beliefs ("simpatia") (20)	Asthma
Family – Not Identified			
Unidentified species – Hummingbirds, "Beija-flor"	1	Whole animal (2), Nest (5)	Asthma (2), Children's fatigue (5)
Numididae			
*Numida meleagris *Linnaeus, 1758 – Helmeted Guineafowl, "Guiné"	1	Blood mixed with sugar (1)	Pertussis
Phasianidae			
*Gallus gallus domesticus *(Linnaeus, 1758) – chicken, "Galinha preta"	6	Fat (1, 4)	Renal calculus, headache, sore throat, nasal congestion (1), fever (4), general swelling (1)
*Pavo cristatus *Linnaeus, 1758 – Common Peafowl, Pavão	1	Feathers (5)	Fatigue (5)
Struthionidae			
*Struthio camelus *Linnaeus, 1766 – Common Ostrich, "Avestruz"	1	Toasted egg shells (4)	Osteoporosis
Tinamidae			
*Nothura maculosa cearensis *Naumburg, 1932 – Spotted Nothura, "Codorniz"	1	Feathers (10)	Effusion

***Mammals***			
Bovidae			
*Bos taurus *Linnaeus, 1758 – Domestic cattle, "Boi"	1	Marrow (8)	Removal of thorns
*Ovis aries *Linnaeus, 1758 – Sheep, "Carneiro"	5	Fat (1), Suet (1), Horn (18)	Articulation problems, pits, pains, forces the child to speak, to assist children who take longer than usual to start speaking (18), rheumatism, "water in the knee"
Canidae			
*Cerdocyon thous *(Linnaeus, 1766) – Crab-eating Fox, "Raposa"	1	Fat(7)	Back ache, osteoporosis
Caviidae			
*Kerodon rupestris *(Wied-Neuwied, 1820) – Rock cavy, "Mocó"	1	Fat(1), Manure(7)	"Tired sight" (1), Effusion (7)
Dasypodidae			
*Euphractus sexcinctu*s (Linnaeus, 1758) – Six-banded Armadillo, "Tatu-peba"	1	Tail (beliefs) (9)	Deafness
Equidae			
*Equus asinus *Linnaeus, 1758 – Donkey, "burro"	1	Hoof (18)	Avoid pregnancy
Erethizontidae			
*Coendou prehensilis *(Linnaeus, 1758) – Brazilian porcupine, "Espinho de Gandú", "Porco-espinho"	1	Pine (5)	Epilepsy
Mephitidae			
*Conepatus semistriatus *(Boddaert, 1785) – Striped hog-nosed skunk, "Ticaca"	2	Fat (2), meat (2)	Asthma, rheumatism, nervous disturbances

Legend: (1) Rubbed on the affected area; (2) ingestion of the roasted animal (or parts of it); (3) a beverage ("*lambedor*") prepared by mixing medicinal plants with honey; (4) ingestion; (5) tea; (6) beverage ('energetic drink') obtained by mixing 1 egg of *Anas platyrhynchos*, 1 pinch of cinnamon, 4 spoon-fulls of Uruçú honey (a species of stingless bee), and 1 cup of guarana. The drink should be consumed during 9 days; (7) Mixed with coffee; (8) Put inside half of a bell pepper and then placed on the area to be treated; (9) Scratch the ear with an armadillo tail; (10) This material is prepared by mixing feathers with the cupira wax to be used as incense that will emit "medicinal" odors; (11) Similar to the item 10, but the incense is made only with honey; (12) a food to be ingested is prepared from the toasted animal together with white onions and "juá" fruit (*Zizyphus joazeiro *Mart.); (13) Leather from *Caiman latirostris *(Daudin, 1801) is mixed with the fat of *Mesoclemmys tuberculata *(Luederwaldt, 1926) and applied on the offended area; (14) Extracted from the poison glands, and 1 teaspoon is taken (approximately 5 ml) everyday in the morning; (15) It is believed that the feather quill (*calamus*) toasted together with the animal's liver and then eaten will inhibit the consumption of alcoholic beverages; (16) Mixed with the coconut oil (*Cocos nucifera *L.) and applied to the affected area; (17) Toasted and macerated in order to produce a "powder" to be mixed with honey (of any bee) and ingested; (18) The rasps should be ingested; (19) Tea made from a sea-horse together with lavender (a medicinal plant); (20) Local belief that asthma can be cured by letting the patient's left-over food be eaten by a bird.

The number of species mentioned in this study was quite expressive, nearing or exceeding the numbers of species sold for medicinal purposes in studies undertaken in other regions of Brazil. Almeida and Albuquerque [22] registered 19 medicinal species in research carried at in Caruaru, Pernambuco; Silva et al. [24] reported the trade of 18 medicinal animals in Recife, Pernambuco, and Costa-Neto [27] and Andrade and Costa-Neto [47] reported the trade of raw materials derived from 16 species in Feira de Santana, Bahia. In the public markets of Maceió, Alagoas State, Freire [48] encountered 17 reptile species commercialized for medicinal purposes (this author worked exclusively with that group). In work carried out in metropolitan areas of northern and northeastern Brazil, Alves and Rosa [16] reported the use and trade of 50 medicinal animal species in Belém (Para State), 61 in São Luís (Maranhão State), 27 in Teresina (Piauí State), and 28 in João Pessoa (Paraiba State). These results indicate that the use of medicinal animals is widespread in probably all urban areas of Brazil.

Most of the animals cited are species native to the *caatinga *biome (only 6 were domestic), demonstrating the importance of the local fauna to regional zootherapeutic practices. This observation is in agreement with previous studies carried out in Brazil that demonstrated that the diversity of medicinal animals used by human populations is influenced by animal diversity in the regional environment [9,10,16]. A similar situation was observed in an examination of the trade in medicinal plants in Witwatersrand, South Africa, where there was found to be a greater use and trade of species collected in biomes near the public markets and of species common in biomes familiar to the commercial gatherers. These authors concluded that the use and trade of medicinal species tends to be proportional of their availability [49].

Only two of the medicinal species cited, the sea horse *Hippocampus reidi *(Ginsburg, 1933) and the star-fish *Oreaster reticulates *(Linnaeus, 1758), do not occur in the area near Santa Cruz do Capibaribe. The use of an oceanic species in the midst of the semi-arid region indicates existence of trade routes for medicinal animals, a situation previously reported by Alves and Rosa [16] for cities in northern and northeastern Brazil. The existence of trade routes is reinforced by the fact that many of the species registered in this study are known to be commercialized in other cities in the country [22,24,27,48]. The present work also identified two medicinal species (*Struthio camelus *Linnaeus, 1766 and *Nasutitermes macrocephalus *(Silvestri, 1903) not previously documented as being used in Brazil. The toasted egg shells of *S. camelus *are used to treat osteoporosis, and the termite *N. macrocephalus *is used to treat asthma.

The interviewees indicated that the following animal parts/products are used as folk remedies: honey, bee wax, fat, secretions, leather, urine, eggs, feathers, nests, blood, marrow, horns, suet, manure, hooves, spines (from porcupines), and meat. A considerable number of species (17) have multiple therapeutic uses, and were prescribed to treat various ailments. The fat of the *Tupinambis merianae *(Duméril & Bibron, 1839), for instance, is indicated for treating four health problems (wounds, sore throats, earaches, perforations). The fat of *Crotalus durissus *Linnaeus, 1758, is indicated for treating rheumatism, swelling, cancer, bone pain, gastritis, eczema, wounds, and backaches; while the chicken fat of *Gallus gallus domesticus *(Linnaeus, 1758) was mentioned as being useful for treating renal calculi, headaches, throat inflammations, nasal congestion, fevers, and general swelling. Similar usages have been reported in other traditional medicine systems as, for example, the carapace and tail of the armadillo *Dasypus novemcinctus *(Linnaeus, 1758) are used to treat diarrhea, tuberculosis, and whooping cough, and to accelerate parturition in Mexico [26]. The fat, skin, and bile ducts of the land monitor *Varanus bengalensis *(Daudin, 1758) are used for treating piles, rheumatism, burns, and spider and snake bites in India [50]. In Bolivia, products derived from the *Agouti paca *(Linnaeus, 1766) are used as remedies for general body pain, leishmaniasis, snakebites, rheumatism, heart pain, bone pain, liver pain, fever, and to alleviate pain during childbirth [23].

Honey bee products and fats (and/or suet) are the most frequently used natural resources, although there have been no reports emphasizing the intensive use of fats and suet as zootherapeutic medicines. This may be due to the fact that the animals used for medicinal purpose are generally vertebrates with significant amounts of fatty tissue that is easy to obtain, store, and transport.

According to the interviewees, zootherapeutic products are used to treat at least 51 different maladies. The most frequently cited treatments are therapies for the respiratory system – and are mainly related to asthma. A similar situation was described by Costa-Neto [27] with regards to the use of medicinal animals in Feira de Santana, Brazil, where animal-based remedies are frequently used to treat respiratory diseases (asthma and bronchitis). Alves and Rosa [51] likewise pointed out that at a minimum of 113 animal species are used in Brazilian traditional medicine for treating asthma. A similar trend in relation to medicinal plants was observed in Pernambuco, where the two most frequent use-categories referred to gastrointestinal and respiratory diseases [52].

The interviewees described many different ways of preparing and administering animal-based remedies. Hard parts generally were sun-dried, grated, and crushed to a powder, to be administered in teas or eaten during meals. Fats, body secretions, and oils were ingested directly or used as ointments. Zootherapeutic products can be prescribed as single-ingredient drugs or mixed with other ingredients. In mixtures, several to many of the more valuable medicinal animal components are mixed with other more available medicinal animal or plant ingredients in more liberal quantities.

The use of some medicinal animals is associated with popular beliefs locally known as '*simpatias*'. These '*simpatias*' are often secretive in nature, so that the people receiving the treatment cannot know what that they are taking, otherwise the remedy will not be effective. This popular belief is commonly associated with the use of medicinal animals in Brazil [11]. Other interesting examples recorded in present study are associated with the use of the tail of *Euphractus sexcinctus*'s (Linnaeus, 1758) to cure deafness – by simply scratching the ear with the tail; and the use of a living specimen of *Cyanocorax cyanopogon *(Wied-Neuwied, 1821) to ingest left-over food from an asthmatic patient, who will then be cured, according to a local belief. This observation is in line with information provided by Alves et al. [11] who pointed out that traditional Brazilian medicine is often associated with local belief systems of *simpatias*, and these popular beliefs may have different implications depending on the manner in which the animal species are used (either dead or alive) and the community's traditions.

Some of the medicinal species cited by interviewees in Santa Cruz do Capibaribe have also been recorded in more distant regions. The honey of *Apis mellifera *(Linnaeus, 1758), for example, is indicated for treating coughs in Brazil and is used for the same purpose in Sudan [53]. Lev [1] reported that the honey of *Apis mellifera *(Linnaeus, 1758) is used in the traditional medicine of Israel as a purgative, and to treat eye inflammations, sore throats, burns, and coughs. Honey from *Melipona scutellaris *Latreille, 1811, was mentioned as a treatment for cancer, fatigue, sexual problems, coughs, and general weakness. This same product was also reported by Costa-Neto [54] as being used as a tonic by residents of Bahia State, Brazil. In Paraiba State in northeastern Brazil, the fat of *Iguana iguana *(Linnaeus, 1758) is used for treating wounds in the local traditional of veterinary medicine [55]. *Ovis aries *(Linnaeus, 1758), a species broadly utilized by the population of Santa Cruz for treating various illnesses, was also previously reported by Costa-Neto and Oliveira [56], Almeida and Albuquerque [22] and Alves and Rosa [9,10]. These examples confirm the knowledge and use of zootherapeutics in different parts of Brazil and around the world.

The present study also noted that some endangered species of medicinal animals (e.g. *H. reidi*) are widely traded. As such, the social use of the biodiversity in mega-diverse countries such as Brazil is crucial to considerations of conservation biology, public health policies, sustainable management of natural resources, and biological prospecting [10] – and there is a great need to stimulate local populations, herbalists, and medicinal animal merchants to adopt conservation measures that avoid over-exploitation, so that the use of these species will not lead to their extinction and the permanent loss of access to their medicinal products.

## Conclusion

Our studies indicated that 37 medicinal animals were being traded in the public markets of Santa Cruz do Capibaribe, and that the zootherapeutic products extracted from them are used to treat 51 ailments – thus indicating the very rich ethnomedical knowledge of the local population in relation to zootherapy. Zootherapy represents an alternative to official medicinal practices in the semi-arid region of northeastern Brazil, and has also become part of urban popular medicine. The local fauna, folk cultures, and the commercial value of these activities are key factors in maintaining and driving the use and commerce of animal species for therapeutic purposes. The lack of monitoring programs or any sort of regulation of this industry in troublesome, however, and argues for undertaking multidisciplinary studies to investigate the social, cultural, economic, clinical, and environmental aspects of these activities to increase our understanding of the use of these medicinal animals and help establish workable management strategies to conserve these zootherapeutic resources.

## Competing interests

The authors declare that they have no competing interests.

## Authors' contributions

RRNA, AV, RRDB and WMSS – Writing of the manuscript, literature survey and interpretation; HNL and MCT- Ethnozoological data, literature survey and interpretation; RRNA and AV – Analysis of taxonomic aspects. All authors read and approved the final manuscript.

## Pre-publication history

The pre-publication history for this paper can be accessed here:


